# ‘He or she maybe doesn't know there is such a thing as a review’: A qualitative investigation exploring barriers and facilitators to accessing medication reviews from the perspective of people from ethnic minority communities

**DOI:** 10.1111/hex.13482

**Published:** 2022-04-05

**Authors:** Anna Robinson, Laura Sile, Thorrun Govind, Harpreet Kaur Guraya, Nicola O'Brien, Vicki Harris, Guy Pilkington, Adam Todd, Andy Husband

**Affiliations:** ^1^ School of Pharmacy, Faculty of Medical Sciences Newcastle University Newcastle upon Tyne UK; ^2^ Population Health Sciences Institute Newcastle University Newcastle upon Tyne UK; ^3^ School of Pharmacy, Faculty of Medical Sciences Liverpool John Moores University Liverpool UK; ^4^ Chair of the English Pharmacy Board Royal Pharmaceutical Society London UK; ^5^ Whale Hill Pharmacy Middlesbrough UK; ^6^ Department of Psychology Northumbria University Newcastle upon Tyne UK; ^7^ Connected Voice Haref Higham House Newcastle upon Tyne UK; ^8^ West End Family Health Primary Care Network Newcastle upon Tyne UK

**Keywords:** ethnic minority, ethnicity, health inequalities, medicine services, medicines review, qualitative

## Abstract

**Introduction:**

Regular reviews of medications, including prescription reviews and adherence reviews, are vital to support pharmacological effectiveness and optimize health outcomes for patients. Despite being more likely to report a long‐term illness that requires medication when compared to their white counterparts, individuals from ethnic minority communities are less likely to engage with regular medication reviews, with inequalities negatively affecting their access. It is important to understand what barriers may exist that impact the access of those from ethnic minority communities and to identify measures that may act to facilitate improved service accessibility for these groups.

**Methods:**

Semi‐structured interviews were conducted between June and August 2021 using the following formats as permitted by governmental COVID‐19 restrictions: in person, over the telephone or via video call. Perspectives on service accessibility and any associated barriers and facilitators were discussed. Interviews were audio‐recorded and transcribed verbatim. Reflexive thematic analysis enabled the development of themes. QSR NVivo (Version 12) facilitated data management. Ethical approval was obtained from the Health Research Authority (ref: 21/HRA/1426).

**Results:**

In total 20 participants from ethnic minority communities were interviewed; these participants included 16 UK citizens, 2 refugees and 2 asylum seekers, and represented a total of 5 different ethnic groups. Three themes were developed from the data regarding the perceived barriers and facilitators affecting access to medication reviews and identified approaches to improve the accessibility of such services for ethnic minority patients. These centred on (1) building knowledge and understanding about medication reviews; (2) delivering medication review services; and (3) appreciating the lived experience of patients.

**Conclusion:**

The results of this study have important implications for addressing inequalities that affect ethnic minority communities. Involving patients and practitioners to work collaboratively in coproduction approaches could enable better design, implementation and delivery of accessible medication review services that are culturally competent.

**Patient or Public Contribution:**

The National Institute for Health Research Applied Research Collaboration and Patient and Public Involvement and Engagement group at Newcastle University supported the study design and conceptualization. Seven patient champions inputted to ensure that the research was conducted, and the findings were reported, with cultural sensitivity.

## 1 INTRODUCTION

Regular reviews of medication are vital to support medicine effectiveness and prescribing safety.^1^, ^2^, ^3^ Previously, medication reviews have been defined as ‘a structured, critical examination of a patient's medicines with the objective of reaching an agreement with the patient about treatment, optimizing the impact of medicines, minimizing the number of medication‐related problems, and reducing waste’.^4^, ^5^ The Royal Pharmaceutical Society of the United Kingdom (UK) deems that conducting medication reviews is a key role for pharmacists and other appropriately trained members of the multidisciplinary team, including doctors and allied health professionals.^4^ In doing so, medication review services address medicine optimization and adherence issues, as well as potentially improve the clinical effectiveness of medicines being taken.^4^, ^6^, ^7^, ^8^, ^9^ This study focuses on medication review services offered by healthcare professionals in the UK working in a primary care setting (e.g., a general practice surgery or community pharmacy), including prescription reviews and adherence and compliance reviews, rather than clinical medication reviews (which require access to clinical information and thus occur more readily in secondary care settings) or medicine use reviews (which have been discontinued).^10^ Medication reviews may take the form of ad‐hoc interventions, with a medication eligible for a New Medicines Service, or aligned with annual long‐term condition reviews.^11^, ^12^ These medication review services may differ from those in other countries or healthcare settings, for example, Australian Home Medication Reviews^13^ or Swiss Polymedication Checks.^14^, ^15^ Optimization of patient outcomes is an underpinning goal of all medication review services; however, inequalities affecting accessibility have been identified, particularly relating to ethnic minority communities.^16^, ^17^, ^18^

A multitude of factors have been identified as contributors of health inequalities amongst ethnic minority populations, including lower health literacy levels, lower socioeconomic status and a greater incidence of ill‐health.^19^, ^20^, ^21^, ^22^, ^23^ The COVID‐19 pandemic further highlighted these inequalities, particularly service accessibility.^24^, ^25^, ^26^ Despite reporting poorer general health when compared to their white counterparts,^27^ and despite being more likely to report a long‐term illness that requires medication,^28^ individuals from ethnic minority backgrounds are less likely to engage in regular medication reviews.^29^, ^30^ Despite the associations between accessibility inequalities and ethnicity, people from ethnic minority communities continue to remain underrepresented participants in health and social care research.^30^, ^31^ These findings can be demonstrated in high‐income countries^27^, ^32^ including the United States of America, where financial burdens of health insurance and remotely delivered consultations during the COVID‐19 pandemic have been reported barriers.^33^, ^34^, ^35^ Evidence suggests that these inequalities also exist for medication reviews; this study sets out to extend the evidence on ethnic inequalities and accessibility in the context of medication reviews. When considering the value that medication reviews can offer in optimizing a person's medication, it is important to (i) understand what barriers may exist that impact the access of those from ethnic minority communities and to (ii) identify measures that may facilitate improved service accessibility for these groups.

## 2 METHODS

### 2.1 Recruitment and sampling

The consolidated criteria for reporting qualitative research (COREQ) checklist was followed (File S1).^36^ This study was conducted during the COVID‐19 pandemic, and social‐distancing restrictions were followed. A blended strategy was used for participant recruitment and data collection, given the capabilities for digital technologies to support qualitative research. Recruitment was conducted using social media (on the professional Twitter accounts of the researchers, Newcastle University School of Pharmacy and Connected Voice), advertising in one general practice surgery (posters in two waiting rooms and a clinician [G. P.] acting as a gatekeeper to introduce the project with eligible participants) and through dissemination to community leaders by charities based in the North East of England (including Connected Voice). All interested participants who contacted the research team were emailed an information sheet and consent form detailing the purpose and aim of the research. Those who wished to participate gave their written consent and were enroled. Study materials including the social media advert, poster, participant information sheet and consent form were translated into different languages to support inclusivity in the research process (these reflected the languages spoken by the ethnic minority groups residing in the research area, including Bengali, Polish, Punjabi, Mandarin, Romanian and Urdu); all documents were reviewed and approved in the ethical approval process. No relationship was established between the researcher and participants before study commencement or recruitment. Inclusion criteria were as follows: participants over 18 years of age who self‐identified as being from an ethnic minority background and living in the North East of England; who took one (or more) regular prescription medication(s); and who had the capacity to consent to taking part in the study. There was no requirement to communicate in the English language; interpreters were involved if required. Purposive sampling was used to recruit participants from different ethnic minority groups reflective of the communities living in the area; participants were of mixed age ranges and had varying sociodemographic and immigration backgrounds (including UK citizens, those with visas and those who were seeking asylum).

### 2.2 Semi‐structured interviews

Semi‐structured interviews were conducted by one researcher (A. R., a female doctoral researcher with qualitative research experience) between June and August 2021. Interviews were conducted via video‐call (Zoom®), telephone call or in person; all participants were offered the choice of their preferred format. Interpreters were available as required, to support translation needs. The interview topic guide (File S1) was developed based on three pilot interviews and covered issues identified in previous research,^29^, ^37^ including participants' experiences of taking medicines; their understanding of medication reviews; their experiences of engaging with these reviews (either in the UK or in their home country); their perspectives of accessing medication reviews; potential barriers or facilitators that affect access; and recommendations for ways to address or improve on challenges.^38^ The topic guide was also informed by the lived experiences of patient champions involved in this study (L. S., T. G. and H. K. G.). For the purposes of this study, the exploration of medication review services will include those delivered by pharmacists, as well as other healthcare professional groups.

### 2.3 Data analysis

All interviews were audio‐recorded to enable data analysis. The audio files were encrypted and transferred electronically (via an electronic, password‐protected drop box) to an external company to be transcribed verbatim. Interpreters were used in some interviews to facilitate three‐way communication between the researcher, the participant and the interpreter; in these instances, the transcripts included the questions asked in English by the researcher and the answers provided by the patient, which were translated into English by the interpreter. All interview data were anonymized during transcription, and all transcripts were checked for accuracy by one researcher (A. R.). For those interviews that required an interpreter, audio accuracy checks were performed by members of the research team (H. K. G., L. S. and T. G.) to ensure that translations were reflective of the spoken content. Participants did not provide comment on the transcripts or feedback on the results.

Following reflexive thematic analysis processes, the principle of constant comparison guided an iterative process of data collection and analysis.^39^, ^40^ Reflexive thematic analysis was performed by two researchers (A. R. and A. H.). Close and detailed reading of the transcripts enabled data familiarization. Initial descriptive codes were identified across the data sets; these were then sorted into coding patterns, which enabled the development of analytic themes. The themes were reviewed, refined and named once coherent and distinctive. Two authors (A. R. and A. H.) performed the data analysis through discussion and, if agreement was not reached, by consensus with the wider research team (N. O., A. T. and V. H.). Interview field notes enhanced this reflective process. NVivo (version 12) software was used to facilitate data management. Given the research timeline and the implications of the COVID‐19 pandemic (further discussed in study limitations), data sufficiency was reached after 18 interviews and thus, study recruitment stopped following interview number 20.^41^ Nonidentifiable pseudonyms (Participant 1, Participant 2) are used throughout to ensure confidentiality.

### 2.4 Considerations when reporting participant demographics

Collection of data on a person's ethnic group is complex, as ethnicity is a multifaceted phenomenon.^42^ There is a lack of consensus on what constitutes an ethnic group when, often, it is something that is self‐defined and subjective to the individual.^43^, ^44^, ^45^ Efforts were made to report a multitude of factors (including a person's first language, religion and citizenship status) to demonstrate the layers that accompany discussions about ethnicity. The UK Office of National Statistics ‘Ethnic group, national identity and religion’,^44^ the UK Census Reporting Classification^46^ and the National Institutes of Health (NIH) ‘Racial and Ethnic Categories and Definitions for NIH Diversity Programmes and Other Reporting Purposes’^45^ guides informed the reporting of participant ethnicity for this study (see Table 1). Data reported also include a column for self‐identified ethnicity and is recorded verbatim from participant interviews.

**Table 1:** Participant demographics

No.	Sex (M/F)	Age (years)	Ethnicity (as per guidance for ethnicity reporting)^44^	Ethnicity (self‐identified by participant, reported verbatim)	Interview format	Interpreter required (Y/N)	Interview duration	First language	Time living in England	Religion	Citizenship status at time of the interview
1	F	55	Asian or Asian British	‘Punjabi Indian’	In person	N	65 min 23 s	Punjabi	29 years	Sikh	UK citizen
2	M	72	Asian or Asian British	‘British Indian’	In person	N	48 min 52 s	Punjabi	54 years	Sikh	UK citizen
3	F	52	Asian or Asian British	‘Indian’	In person	Y	45 min 10 s	Punjabi	21 years	Sikh	UK citizen
4	M	31	Asian or Asian British	‘Malaysian Chinese’	Video call	N	43 min 47 s	Mandarin	9 years	Buddhist	UK citizen
5	M	75	Asian or Asian British	‘British Asian Pakistani’	Video call	N	60 min 3 s	Urdu	35 years	Atheist	UK citizen
6	M	70	Asian or Asian British	‘Chinese’	In person	Y	49 min 35 s	Mandarin	20 years	Christian	UK citizen
7	F	67	Asian or Asian British	‘Chinese’	In person	Y	51 min 11 s	Mandarin	12 years	Christian	UK citizen
8	F	26	Black, African Caribbean or Black British	‘Black British’	Video call	N	42 min 25 s	English	26 years	Atheist	UK citizen
9	F	60	Black, African Caribbean or Black British	‘Black British’	Telephone	N	62 min 16 s	English	21 years	Muslim	UK citizen
10	M	68	Black, African Caribbean or Black British	‘British Nigerian’	Telephone	N	45 min 11 s	English	21 years	Muslim	UK citizen
11	F	65	White	‘British’	In person	N	62 min 21 s	English	65 years	Jewish	UK citizen
12	M	50	White	‘White other’	Telephone	N	43 min 20 s	Hungarian	14 years	Atheist	UK citizen
13	F	47	White	‘White other’	Telephone	N	53 min 45 s	Hungarian	13 years	Christian	UK citizen
14	F	33	Other ethnic group	‘Arab ethnicity’	Video call	N	52 min 21 s	Arabic	3 years	Muslim	Residency visa
15	F	34	Other ethnic group	‘Arab’	Telephone	N	50 min 53 s	Arabic	3 years	Muslim	Residency visa
16	F	38	Other ethnic group	‘Arab’	Telephone	Y	61 min 7 s	Arabic	3 years	Muslim	Asylum seeker
17	M	65	Other ethnic group	‘Israeli’	In person	N	62 min 21 s	Hebrew	45 years	Jewish	UK citizen
18	F	39	Mixed or multiple ethnic groups	‘Mixed—Arab and Turkish’	Telephone	N	55 min 30 s	Arabic	8 years	Muslim	Asylum seeker
19	F	34	Mixed or multiple ethnic groups	‘White Indo‐Caribbean’	Video call	N	37 min 41 s	English	24 years	Atheist	UK citizen
20	M	55	Mixed or multiple ethnic groups	‘Mixed British and Arab’	Video call	N	49 min 30 s	English	41 years	Atheist	UK citizen

Abbreviations: F, female; M, male; min, minutes; N, no; s, seconds; UK, United Kingdom; Y, yes.

### 2.5 Ethical approval

Ethical approval was obtained from the NHS Health Research Authority (HRA) and Care Research Wales (ref: 21/HRA/1426), and research governance was granted by the participating NHS organizations.

## 3 RESULTS

### 3.1 Participant demographics

In total, 20 participants were recruited, including 16 UK citizens, 2 asylum seekers and 2 people in receipt of residency visas. Participant characteristics are described in Table 1, and there were no refusals to partake, participant dropouts or repeat interviews. The average age of the participants was 52 years (SD: 15.3), and five different ethnic groups were represented within the sample. Seven interviews were conducted in person, seven over the telephone and six were conducted using the video call‐based software, Zoom®. Interview durations ranged between 37 and 65 min (average: 52 min [SD: 7.8]). Fourteen of the participants reported their first language as one other than English and four participants required an interpreter to aid discussions, covering Punjabi, Arabic and Mandarin languages; in all instances, family members acted as interpreters.

**Figure 1:**
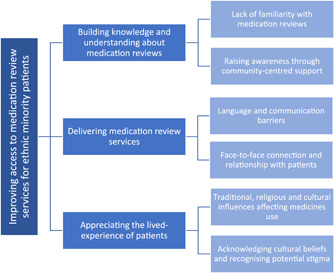
Factors affecting the accessibility of medicine review services for ethnic minority patients

Three overarching themes were developed from the data to highlight factors that affected access to medication review services for ethnic minority patients. The themes centred on (1) building knowledge and understanding about medication reviews; (2) the delivery of medication review services; and (3) appreciating the lived experience of patients (Figure 1). Each theme and subtheme is discussed in turn, using anonymized verbatim interview quotes to reflect patient perspectives.

### 3.2 Theme 1: Building knowledge and understanding about medication reviews

In all interviews, participants discussed challenges relating to awareness of medication reviews within ethnic minority communities; many reported being unaware of medication review services, which, understandably, impacted on their access. Reasons for this included a lack of knowledge and familiarity with the medication review process in the UK compared to that of their home country, as well as not understanding the benefits of the process. The use of peer‐support networks and community signposting were discussed as potential strategies to overcome this.

#### 3.2.1 Lack of familiarity with medication review

Participants spoke of unfamiliarity with medication review processes, with one participant stating ‘I got this feeling a lot of people doesn't really know what service the GP practice and the pharmacy do offer for medicines… He or she maybe doesn't know there is such a thing as a medicine review’ (Participant 4). Another discussed never having a medication review in their home country and, as a result, did not understand why it was required when living in the UK.

*When you come from India, you don't know that you need the (medicine) review. I just get medicines in India… no questions if they working… then the lady pharmacist here, she tells me I need one and I think, why?… But in England, it is different with having the review, then you learn to know much more and learn how such‐and‐such medicine works… you can check these things every year time, which I like better and I understand much more of the medicines now*. (Participant 3)

Healthcare professionals involved in the delivery of medication reviews were recognized as integral in supporting patient understanding. Participants discussed the need to educate professionals of ways to help overcome the possible patient‐knowledge barriers to improve access.

*(Healthcare professionals) maybe not know that we don't have this in our own country before… they maybe explain it better for us… then we can know to understand (the medicines review) is existing and why it is good for us patients to have it*. (Participant 3)

Participants viewed better advertising of medication reviews as a vital first step in supporting asylum seeker and refugee groups upon their arrival to a new country. Medicine‐focused materials could be provided at this critical timepoint ‘when someone is at their most vulnerable, they need to know where to go for these things… making sure (the medications) are prescribed, making sure they are safe – that's all important’ (Participant 19).

#### 3.2.2 Raising awareness through community‐centred support

Participants identified facilitators that could build awareness of medication review services within communities; by involving leaders from their community or religious groups, access to medication review services could be improved. Two participants discussed how their Rabbi (a qualified pharmacist) could ‘indicate to the Jewish community, (medication reviews) is something we could be having to look after ourselves in a specific medicines‐way… (whilst) adhering to the principles of our religion’ (Participant 11). Similarities were discussed by Muslim and Sikh participants, believing that religious leaders could raise awareness of medication reviews. Signposting was also viewed as a strategy to build community engagement, including the placement of flyers in ‘the mosques and temples’ to advertise ways to access medication reviews and to list ‘all of the local pharmacies where they could actually go’ (Participant 8). Alternatively, ‘community gatherings, not necessarily in a place of worship’ were recognized as valuable in engaging members of the community who may not follow a religion (Participant 8). Another participant recognized that diverse geographical areas may hold diversity events that ‘could be useful because you'd have people from different groups there… it's about all‐community information for minorities’ (Participant 19).

Digital peer support was also discussed, such as community WhatsApp® messaging groups where peers could message about queries, including how to access pharmacies for prescriptions or purchasing medicines and ‘where the nearest chemist (pharmacy) was’ (Participant 5). Participants also described using WhatsApp® to support with translation needs.

*I am member of several WhatsApp® groups (where)… people who need the help, they explaining the problem in Arabic and me and my friends translate to the right words so they can explain to pharmacist*. (Participant 15)

### 3.3 Theme 2: Delivering medication review services

The way in which medication review services are *delivered* was recognized as a priority when overcoming accessibility‐related barriers. Participants placed emphasis on addressing language and communication barriers between the healthcare professional and the patient. Many discussed the ongoing impact on communication resulting from the COVID‐19 global pandemic. Building patient–provider relationships through face‐to‐face services was perceived as a facilitator supporting access to medication reviews.

#### 3.3.1 Addressing language and communication barriers

Language and communication were raised as major barriers affecting people's access to obtaining medicine‐specific advice. Fourteen participants reported that English was not their first language, and four participants contributed by using interpreters. Interviewees discussed the barriers and safety implications ‘if someone is not a native English speaker and suddenly being expected to take all of this medication and all this jargon of names… as well as being expected to know what (the healthcare professional) mean when they give out advice’ (Participant 10). If medication review services were delivered in a way that ‘took the language barrier into consideration’, participants believed that people may be ‘more inclined to attend and ask information… to start learning the ins and outs of what our medications are for and why we are on them’ (Participant 4). These perceptions linked to creating positive medicine‐encounters, helping ‘(ethnic minority patients) to get comfortable with the idea of taking (their medicines) and asking advice to keep them safe’ (Participant 4).

Reducing feelings of vulnerability ‘if you don't speak the language’ was essential to continue creating positive medicine‐encounters during medication consultations (Participant 8). Interpreters were discussed as facilitators of improved access by ‘translating all of the discussion back to the patient's first language so they're involved… so they feel involved and listened to’ (Participant 19). One participant, who was a refugee at the time of the interview, reflected on the lack of availability of interpreters in pharmacy settings, which limited their ability to have medication discussions with a pharmacist (Participant 16). Another reflected on their experience of translating for a ‘new arrival’ refugee family, who may not have attended for medication advice if they had not had an interpreter (Participant 15).

*I know they would not have gone (to seek medication advice) if they did not have me for the translating… I know that the people in the Arabic community will leave to suffer in silence rather than speaking up… it is why translating the language gap is so important*. (Participant 15)

Non‐verbal communication strategies, including the translation of written text into other languages, were acknowledged as facilitators to engagement with medication review services. One participant remarked ‘“why can't they put things on the labels in Urdu for me?” so it can be easier for reading it’ (Participant 15). Another discussed how the translation of medication safety messages could promote and facilitate ‘starting to have the conversations with the vulnerable people who don't speak English well… (in order to)make them aware of the safety of the medicines… that they take the safe medicines at the safe doses’ (Participant 14). Alongside this, verbal translations were recognized as a way of meeting the needs of ‘people who speak languages that are spoken only, where they cannot be written down’ (Participant 17).

#### 3.3.2 Face‐to‐face connections and reassurance

Forming a relationship between participants and healthcare professionals, through face‐to‐face appointments, was deemed to be an important facilitator to accessing services. One participant described a lack of connection when reviewing their medicines remotely, reporting ‘it's not the same when you talk on the phone… (the pharmacist) want to discuss with you what is the medicine, why you are on the medicine, is it helpful? But sometimes I need to point to the box, want to describe my answer like that, or want to point to the things, but I cannot if it is talking on the phone… like my legs when I have the swelling in my ankles’ (Participant 1).

Experiences of consultations during the COVID‐19 pandemic were discussed. One participant described the benefits of in‐person consultations to help with nonverbal communication and body language, as ‘sometimes people not say anything, their face tells you. It is harder to read someone when you not in the place with them… I think it's the facial expressions, maybe the reactions, maybe the body language’ (Participant 3, via an interpreter). Two participants discussed challenges that arose with mask‐wearing, describing ‘these masks, they are making a problem for people too, as wearing a mask is very difficult to communicate’ (Participant 3, via an interpreter) and ‘I need to watch people's mouths move… it's easier to understand that way if English is not your first language like me’ (Participant 12).

### 3.4 Theme 3: Appreciating the lived experience of patients

Many participants felt it important for healthcare professionals to appreciate the lived experience of ethnic minority people within their patient population. Participants recognized how cultural or religious traditions may affect a person's relationship and behaviour with medicines, including dietary practices. Participants also reflected on the potential for cultural stigma to impact access and, in turn, viewed cultural awareness and cultural competency training for healthcare professionals as a facilitator to overcome barriers to medication reviews.

#### 3.4.1 Traditional, religious or cultural influences that affect medicine use

Participants discussed the importance of healthcare professionals appreciating a patient's culture or religion; one participant, seeking asylum at the time of interview, explained that if the clinical focus of the healthcare professional does not align with the religious or cultural focus of the person taking the medicine, it could become a barrier to a person's engagement.

*The doctor focus only on the symptoms and the suitable medicine for helping me, but no one focuses on the medicine, if it is related to certain foods, then this can be a big problem for Muslim culture and Muslim religion… we might not take it, we might not want to discuss further with them… that is our beliefs*. (Participant 16, via an interpreter)

Participants discussed the importance of cultural sensitivity shown by the healthcare professional as religious beliefs and practices may influence medicine use and engagement with medication review services. One participant explained that information is ‘not translated on the labels on the medicine or mentioning by the doctor or pharmacist that medicines like this have the alcohol content… I am not allowed to take any alcohol at all as a Muslim and this is very, very important to me’ (Participant 16, via an interpreter). Another described feelings of generalization when healthcare professionals failed to recognize their religious behaviours as a Sikh, in comparison to those of Muslim faith.

*Sometimes (healthcare professionals) don't understand – I told them I'm a Sikh and I'm an Indian background, so he knew I could have the certain medicine that maybe the Muslims can't have with it being not Halal ingredients, but he didn't understand it… to me that is a big, big difference… this is why it is important for us to feel that they (healthcare professionals) respect and know our cultures and our backgrounds*. (Participant 1).

One participant, who reported their ethnicity as Malaysian Chinese, discussed their personal beliefs about use of traditional medicines, in comparison with prescribed medications. They explained ‘it is the ancestor's beliefs; this is very important in the culture of my family… the peoples probably prefer to take something herbal or of the Chinese traditional medicines because they are familiar with it’ (Participant 4). Two other participants reported that they preferred to ‘first try the traditional remedies… only take the medicine if think traditional won't work’ (Participant 7, via an interpreter). Cultural awareness of patient preferences from these communities was viewed as significant.

*The way I feel about asking their opinion with the medicines… if I know they have an idea of my culture then it's a better thing for me… no judgement if I take the herbals*. (Participant 4)

#### 3.4.2 Acknowledging cultural beliefs and recognizing potential stigma

Challenges relating to cultural beliefs and stigma appeared to influence a person's readiness to access the diagnosis and treatment of certain medical conditions, most often mental health conditions. One participant, a member of the Orthodox Jewish community, discussed barriers to access for patients with ‘any sort of mental health issues’ as ‘Jewish people are very, very closed about (mental health conditions)… when they come to marry, some people will be concerned’ (Participant 11). They described how this may impact a person accessing medication reviews in fear of *‘*embarrassment’ amongst the community (Participant 11).

*People will not discuss it (mental health conditions)… especially if the pharmacist was a member of the community himself… that way it might become common knowledge and affect the family's reputation*. (Participant 11)

While similar feelings are likely to also occur in wider communities, one participant contextualized the significance of embarrassment amongst their own cultural group, when taking medication for depression.

*I went to the GP for my medications and discovered that the doctor is Egyptian man. It was not easy … I didn't want him to have access to the list of depression medications I take… it is not something I wish my community (members) to know*. (Participant 15)

Cultural competency training was perceived to facilitate access to medication review services. Participants called for services to ‘have more culturally sensitive’ (Participant 5) underpinnings and acknowledged how ‘some kind of training needs to be given around this’ to act as a potential enabler of culturally appreciative improvement (Participant 18). One participant discussed recommendations including ‘listening, empathy… learn about the person, be mindful of (their past)… accepting more that there could be something in their religion why they do something… like Ramadan and fasting and not taking tablets sometime’ (Participant 18). As part of cultural competency training, and in association with in‐person preferences for consultations, body language and active listening training was recognized as imperative.

*(appreciating) the possible past‐experiences that someone might have, because that isn't something they'll get over quickly. It's something that will be internalised and potentially affect them actually coming to (review their medications) if they know you'll be judgemental or not listen to them… show empathy… (take) time to listen*. (Participant 5)

## 4 DISCUSSION

This study builds on the limited evidence base that considers the accessibility of medication review services for ethnic minority populations living within the UK. This study sheds light on the existing barriers, facilitators and lived experiences of patients and has led to the development of three themes, each regarded as significant in enabling access to medication review services.

The barriers of communication and language are well known in the wider literature associated with inequalities in access to healthcare services amongst ethnic minority groups.^47^, ^48^, ^49^ While interpreters were viewed as facilitators for accessibility, there is evidence to discourage the use of family members or friends in this role; however, availability limitations across care settings like community pharmacy make this a challenge.^50^, ^51^, ^52^, ^53^, ^54^ Multilingual medication labels have also been a documented strategy of improving access to medicines for ethnically diverse populations.^55^, ^56^, ^57^ This study recognized the value of face‐to‐face communication for building trust and relationships, and acknowledged that the COVID‐19 pandemic further exacerbated communication issues.^58^, ^59^, ^60^ Mask‐wearing was perceived as a communication barrier for nonnative English speakers.^61^

Participants from this study placed emphasis on the value of peer‐support networks to overcome accessibility barriers to medication review services, similar to work in empowering ethnic minority groups through community outreach and signposting.^62^ Religious‐ and community‐based settings were discussed as places where medicine services could be advertised, recognizing the value of partnership‐working when promoting health campaigns^63^ to address inequalities in accessibility.^64^ Cooperation between the public, healthcare professionals and community and religious leaders has been associated with empowering disadvantaged groups to access healthcare services.^62^ This approach could cultivate culturally congruent healthcare environments and support the formation of culturally competent relationships between healthcare professionals and communities.^62^, ^65^, ^66^ Success with peer‐led support has been demonstrated pre‐ and postoperatively,^67^, ^68^ when managing long‐term conditions^69^ and in smoking cessation and weight management campaigns.^70^, ^71^, ^72^ However, peer support has previously been associated with the dissemination of medical misinformation.^73^, ^74^ Findings from this review placed emphasis on digital peer support in the form of community WhatsApp® groups and supported their use and acceptability.^75^ Research should seek to gain further insight into digital interventions that may facilitate access to medication reviews for ethnic minority patient groups.

Participants identified the value in codesign alongside policy‐makers and healthcare providers when shaping future service design to improve the delivery of culturally competent medication review services.^76^ Previous work identified the potential of pharmacy‐based codesign approaches when engaging with marginalized groups.^77^, ^78^ Enhanced cultural competency of healthcare professionals supports the confrontation of inequalities in marginalized populations.^79^, ^80^, ^81^ Understanding medication‐related needs of ethnic minority communities, and the possible associated religious or cultural practices, may support greater appreciation of factors that influence medicine use.^79^, ^82^ Cultural competence training should be implemented in pharmacy curricula to widen knowledge of cultures within the populations they treat.^83^ While this study offers insight into improving access to medication services, there remains a knowledge gap evaluating the extent to which addressing these factors results in improved health outcomes for ethnic minority groups in the long term. Collaborative work could support the development and refinement of a targeted medicine review intervention to support better access to medication reviews.^29^, ^30^, ^77^ To further investigate the barriers and facilitators that would enable improved access to medication review services, future studies should seek to adopt codesign approaches to develop inclusive services that meet the needs of the communities. Consideration of this is also required for private healthcare settings, such as those in the United States, where financial reimbursement for such services may prove to be an additional barrier to their implementation.^84^

The National Institute for Health Research (NIHR) toolkit and guidance from the NIHR INCLUDE project were used to support participant recruitment.^85^, ^86^ Members of the research team undertook cultural competency training delivered by the NIHR and Connected Voice.^86^ Seven patient champions, who were representatives of the communities involved, were appointed to the research steering group and provided input ensuring cultural sensitivity throughout the research process, three of whom are listed as coauthors (L. S., T. G. and H. K. G.). The voices of numerous ethnic minority groups are included in this study; 20 participants of mixed ethnicities and age ranges were purposively sampled and interviewed. However, the authors acknowledge that there are some limitations with this study. The number of participants holding refugee and asylum‐seeker status (*n* = 4) was outweighed by those who held UK Citizenship status (*n* = 16). The COVID‐19 pandemic impacted participant recruitment, particularly with refugee and asylum‐seeker groups, given funding and staffing issues on a charity level. The intended in‐person data collection was also impacted; although 7 interviews took place in person, the remaining 13 used remote approaches. While remote interviewing holds many benefits,^87^, ^88^, ^89^ user familiarity with video call software may have impacted its wider adoption amongst the study participants^90^; although no differences were noted in the data collected across the different interview formats.^89^ Family members supported as interview interpreters in this study; the authors acknowledged the potential limitations of this, given that the discussion was centred around medicines and health.^91^, ^92^ The focus of this study was ethnic minority populations living in the UK; thus, findings may not be generalizable in other countries. However, facilitating access is fundamental and must be considered to overcome global accessibility inequalities for ethnic minority communities.

## 5 CONCLUSION

This study provides much‐needed evidence of the barriers and facilitators that affect access to medication review services for people from ethnic minority communities. The results have important implications for overcoming ethnic inequalities. The data highlighted the significance of raising awareness of the medicine review services and understanding each person's lived experiences to address barriers that currently affect access. Delivering medication review services with cultural competency is vital; steps should be taken to address potential language barriers and build patient–provider relationships through in‐person medication reviews. Collaborative coproduction approaches could enable better design, implementation and delivery of medication services that are accessible and culturally competent to best meet the needs of ethnic minority communities.

## CONFLICTS OF INTEREST

The authors declare that there are no conflicts of interest.

## AUTHOR CONTRIBUTIONS

Anna Robinson was appointed Research Assistant for this project and led on the data collection and writing of this manuscript. Andy Husband and Adam Todd oversaw the running of this project as Principal Investigators and provided project management expertize. NO provided qualitative methodological input and expertize. Guy Pilkington and Vicki Harris supported the recruitment of participants and links to ethnic minority communities. Thorrun Govind, Harpreet Kaur Guraya and Laura Sile contributed in their appointment as patient champions and ensured cultural appropriateness and sensitivity throughout the research process. All authors read, provided comments on and approved the final manuscript.

## Supporting information

Supporting information.

## Data Availability

The data that support the findings of this study are available from the corresponding author upon reasonable request.

## References

[1] Nunes V , Neilson J , O'flynn N , et al. Medicines Adherence: Involving Patients in Decisions About Prescribed Medicines and Supporting Adherence. Royal College of General Practitioners; 2009. 21834197

[2] Clyne B , Bradley MC , Smith SM , et al. Effectiveness of medicines review with web‐based pharmaceutical treatment algorithms in reducing potentially inappropriate prescribing in older people in primary care: a cluster randomized trial (OPTI‐SCRIPT study protocol). Trials. 2013;14(1):1‐12. 23497575 10.1186/1745-6215-14-72 PMC3621288

[3] Jain S , Upadhyaya P , Goyal J , et al. A systematic review of prescription pattern monitoring studies and their effectiveness in promoting rational use of medicines. Perspect Clin Res. 2015;6(2):86. 25878953 10.4103/2229-3485.154005 PMC4394586

[4] Royal Pharmaceutical Society (RPS) . *Medication review—quick reference guide*. Accessed December 1, 2021. https://www.rpharms.com/resources/quick-reference-guides/medication-review2021

[5] National Institute for Health and Care Excellence (NICE) . *Medicines optimisation: the safe and effective use of medicines to enable the best possible outcomes—NICE Guideline NG5*. Accessed December 1, 2021. https://www.nice.org.uk/guidance/ng5/chapter/1-Recommendations2015 26180890

[6] Europe PCN . Position paper on the PCNE definition of medication review. 2016. Accessed December 1, 2021. https://www.pcne.org/upload/files/149_Position_Paper_on_PCNE_Medication_Review_final.pdf

[7] Clyne W , Blenkinsopp A , Seal R . *A guide to medication review*. NPC. 2008.

[8] Goh BQ , Tay AHP , Khoo RSY , Goh BK , Lo PFL , Lim CJF . Effectiveness of medication review in improving medication knowledge and adherence in primary care patients. Proc Singapore Healthc. 2014;23(2):134‐141.

[9] de Oliveira Santos Silva R , Macêdo LA , Dos Santos Júnior GA , Aguiar PM , de Lyra DP . Pharmacist‐participated medication review in different practice settings: service or intervention? An overview of systematic reviews. PLoS One. 2019;14(1):e0210312. 30629654 10.1371/journal.pone.0210312 PMC6328162

[10] Bulajeva A , Labberton L , Leikola S , et al. Medication review practices in European countries. Res Soc Adm Pharm. 2014;10(5):731‐740. 10.1016/j.sapharm.2014.02.005 24661800

[11] Holland R , Desborough J , Goodyer L , Hall S , Wright D , Loke YK . Does pharmacist‐led medication review help to reduce hospital admissions and deaths in older people? A systematic review and meta‐analysis. Br J Clin Pharmacol. 2008;65(3):303‐316. 18093253 10.1111/j.1365-2125.2007.03071.x PMC2291244

[12] Hasan Ibrahim AS , Barry HE , Hughes CM . A systematic review of general practice‐based pharmacists' services to optimize medicines management in older people with multimorbidity and polypharmacy. Fam Pract. 2021;38(4):509‐523. 33506870 10.1093/fampra/cmaa146

[13] Swain L , Barclay L . Medication reviews are useful, but the model needs to be changed: perspectives of Aboriginal Health Service health professionals on home medicines reviews. BMC Health Serv Res. 2015;15(1):366. 26357987 10.1186/s12913-015-1029-3 PMC4566399

[14] Messerli M , Vriends N , Hersberger KE . Humanistic outcomes and patient acceptance of the pharmacist‐led medication review “Polymedication Check” in primary care in Switzerland: a prospective randomized controlled trial. Patient Prefer Adherence. 2018;12:1071. 29950820 10.2147/PPA.S160789 PMC6016257

[15] Metaxas C , Albert V , Habegger S , Messerli M , Hersberger KE , Arnet I . Patient knowledge about oral anticoagulation therapy assessed during an intermediate medication review in Swiss community pharmacies. Pharmacy. 2020;8(2):54. 10.3390/pharmacy8020054 PMC7355591 32231095

[16] Aspinall PJ , Jacobsen B . Ethnic Disparities in Health and Health Care: A Focused Review of the Evidence and Selected Examples of Good Practice: Executive Summary. London Health Observatory; 2004.

[17] Cooper LA , Hill MN , Powe NR . Designing and evaluating interventions to eliminate racial and ethnic disparities in health care. J Gen Intern Med. 2002;17(6):477‐486. 12133164 10.1046/j.1525-1497.2002.10633.x PMC1495065

[18] Nelson A . Unequal treatment: confronting racial and ethnic disparities in health care. J Natl Med Assoc. 2002;94(8):666. 12152921 PMC2594273

[19] Chauhan A , Walton M , Manias E , et al. The safety of health care for ethnic minority patients: a systematic review. Int J Equity Health. 2020;19(1):1‐25. 10.1186/s12939-020-01223-2 PMC7346414 32641040

[20] Johnstone M‐J , Kanitsaki O . Culture, language, and patient safety: making the link. Int J Qual Health Care. 2006;18(5):383‐388. 16956931 10.1093/intqhc/mzl039

[21] Bakullari A , Metersky ML , Wang Y , et al. Racial and ethnic disparities in healthcare‐associated infections in the United States, 2009–2011. Infect Control Hosp Epidemiol. 2014;35(S3):S10‐S16. 10.1086/677827 25222888

[22] Suurmond J , Uiters E , de Bruijne MC , Stronks K , Essink‐Bot M‐L . Explaining ethnic disparities in patient safety: a qualitative analysis. Am J Public Health. 2010;100(S1):S113‐S117. 20147688 10.2105/AJPH.2009.167064 PMC2837434

[23] van Rosse F , Suurmond J , Wagner C , de Bruijne M , Essink‐Bot M‐L . Role of relatives of ethnic minority patients in patient safety in hospital care: a qualitative study. BMJ Open. 2016;6(4):e009052. 10.1136/bmjopen-2015-009052 PMC4838722 27056588

[24] Razai MS , Kankam HK , Majeed A , Esmail A , Williams DR . Mitigating ethnic disparities in covid‐19 and beyond. BMJ. 2021:​ 372. 10.1136/bmj.m4921 33446485

[25] Lassale C , Gaye B , Hamer M , Gale CR , Batty GD . Ethnic disparities in hospitalisation for COVID‐19 in England: the role of socioeconomic factors, mental health, and inflammatory and pro‐inflammatory factors in a community‐based cohort study. Brain Behav Immun. 2020;88:44‐49. 32497776 10.1016/j.bbi.2020.05.074 PMC7263214

[26] Pan D , Sze S , Minhas JS , et al. The impact of ethnicity on clinical outcomes in COVID‐19: a systematic review. EClinicalMedicine. 2020;23:100404. 32632416 10.1016/j.eclinm.2020.100404 PMC7267805

[27] Nazroo JY , Falaschetti E , Pierce M , Primatesta P . Ethnic inequalities in access to and outcomes of healthcare: analysis of the Health Survey for England. J Epidemiol Community Health. 2009;63(12):1022‐1027. 19622520 10.1136/jech.2009.089409

[28] Gault I , Pelle J , Chambers M . Co‐production for service improvement: developing a training programme for mental health professionals to enhance medication adherence in Black, Asian and Minority Ethnic Service Users. Health Expect. 2019;22(4):813‐823. 31250521 10.1111/hex.12936 PMC6737759

[29] Latif A , Mandane B , Ali A , Ghumra S , Gulzar N . A qualitative exploration to understand access to pharmacy medication reviews: views from marginalized patient groups. Pharmacy. 2020;8(2):73. 10.3390/pharmacy8020073 PMC7357163 32357462

[30] Latif A , Tariq S , Abbasi N , Mandane B . Giving voice to the medically under‐served: a qualitative co‐production approach to explore patient medicine experiences and improve services to marginalized communities. Pharmacy. 2018;6(1):13. 10.3390/pharmacy6010013 PMC5874552 29382062

[31] Smart A , Harrison E . The under‐representation of minority ethnic groups in UK medical research. Ethn Health. 2017;22(1):65‐82. 27174778 10.1080/13557858.2016.1182126

[32] Nazroo JY , Williams DR . The social determination of ethnic/racial inequalities in health. Soc Determ Health. 2006;2:238‐266.

[33] Chunara R , Zhao Y , Chen J , et al. Telemedicine and healthcare disparities: a cohort study in a large healthcare system in New York City during COVID‐19. J Am Med Inform Assoc. 2021;28(1):33‐41. 32866264 10.1093/jamia/ocaa217 PMC7499631

[34] Schrader CD , Lewis LM . Racial disparity in emergency department triage. J Emerg Med. 2013;44(2):511‐518. 22818646 10.1016/j.jemermed.2012.05.010

[35] Adhikari S , Pantaleo NP , Feldman JM , Ogedegbe O , Thorpe L , Troxel AB . Assessment of community‐level disparities in coronavirus disease 2019 (COVID‐19) infections and deaths in large US metropolitan areas. JAMA Netw Open. 2020;3(7):e2016938‐e2016938. 32721027 10.1001/jamanetworkopen.2020.16938 PMC7388025

[36] Tong A , Sainsbury P , Craig J . Consolidated criteria for reporting qualitative research (COREQ): a 32‐item checklist for interviews and focus groups. Int J Qual Health Care. 2007;19(6):349‐357. 17872937 10.1093/intqhc/mzm042

[37] Latif A , Carter T , Rychwalska‐Brown L , Wharrad H , Manning J . Co‐producing a digital educational programme for registered children's nurses to improve care of children and young people admitted with self‐harm. J Child Health Care. 2017;21(2):191‐200. 29119822 10.1177/1367493517697853

[38] Robinson A , Elarbi M, Todd A, Husband A . A qualitative exploration of the barriers and facilitators affecting ethnic minority patient groups when accessing medicine review services: perspectives of healthcare professionals. Health Expect . 2021. 10.1111/hex.13410 PMC8957739 34951087

[39] Braun V , Clarke V . Reflecting on reflexive thematic analysis. Qual Res Sport, Exerc Health. 2019;11(4):589‐597.

[40] Braun V , Clarke V . What can “thematic analysis” offer health and wellbeing researchers? Int J Qual Stud Health Well‐Being. 2014;9:26152. 25326092 10.3402/qhw.v9.26152 PMC4201665

[41] Malterud K , Siersma VD , Guassora AD . Sample size in qualitative interview studies: guided by information power. Qual Health Res. 2015;26(13):1753‐1760. 10.1177/1049732315617444 26613970

[42] Burton J , Nandi A , Platt L . Measuring ethnicity: challenges and opportunities for survey research. Ethn Racial Stud. 2010;33(8):1332‐1349.

[43] Aspinall PJ . What kind of mixed race/ethnicity data is needed for the 2020/21 global population census round: the cases of the UK, USA, and Canada. Ethn Racial Stud. 2018;41(11):1990‐2008.

[44] Office for National Statistics . Measuring equality: a guide for the collection and classification of ethnic group, national identity and religion data in the UK. 2021. Accessed February 1, 2022.https://www.ons.gov.uk/methodology/classificationsandstandards/measuringequality/ethnicgroupnationalidentityandreligion

[45] National Institutes of Health (NIH) . *Racial and ethnic categories and definitions for NIH diversity programs and for other reporting purposes. * 2015. Accessed February 1, 2022. https://grants.nih.gov/grants/guide/notice-files/not-od-15-089.html

[46] Angelsen A , Lund JF . Designing the household questionnaire. Measuring Livelihoods and Environmental Dependence Methods for Research and Fieldworks. Center for International Forestry Research; 2011:107‐126.

[47] Ahmed S , Lee S , Shommu N , Rumana N , Turin T . Experiences of communication barriers between physicians and immigrant patients: a systematic review and thematic synthesis. Patient Exp J. 2017;4(1):122‐140.

[48] Westberg SM , Sorensen TD . Pharmacy‐related health disparities experienced by non–English‐speaking patients: impact of pharmaceutical care. J Am Pharm Assoc. 2005;45(1):48‐54. 10.1331/1544345052843066 15730117

[49] Watermeyer J . “She will hear me”: how a flexible interpreting style enables patients to manage the inclusion of interpreters in mediated pharmacy interactions. Health Commun. 2011;26(1):71‐81. 21218301 10.1080/10410236.2011.527623

[50] Rosenberg E , Seller R , Leanza Y . Through interpreters' eyes: comparing roles of professional and family interpreters. Patient Educ Couns. 2008;70(1):87‐93. 18031970 10.1016/j.pec.2007.09.015

[51] Brisset C , Leanza Y , Laforest K . Working with interpreters in health care: a systematic review and meta‐ethnography of qualitative studies. Patient Educ Couns. 2013;91(2):131‐140. 23246426 10.1016/j.pec.2012.11.008

[52] Leanza Y , Boivin I , Rosenberg E . Interruptions and resistance: a comparison of medical consultations with family and trained interpreters. Soc Sci Med. 2010;70(12):1888‐1895. 20378224 10.1016/j.socscimed.2010.02.036

[53] Ho A . Using family members as interpreters in the clinical setting. J Clin Ethics. 2008;19(3):223‐233. 19004432

[54] Bellamy K , Ostini R , Martini N , Kairuz T . Access to medication and pharmacy services for resettled refugees: a systematic review. Aust J Prim Health. 2015;21(3):273‐278. 25577397 10.1071/PY14121

[55] Zargarzadeh AH , Law AV . Access to multilingual prescription labels and verbal translation services in California. Res Social Adm Pharm. 2011;7(4):338‐346. 21272528 10.1016/j.sapharm.2010.08.001

[56] Weiss L , Gany F , Rosenfeld P , et al. Access to multilingual medication instructions at New York City pharmacies. J Urban Health. 2007;84(6):742‐754. 17926130 10.1007/s11524-007-9221-3 PMC2232041

[57] Sharif I , Lo S , Ozuah PO . Availability of Spanish prescription labels. J Health Care Poor Underserved. 2006;17(1):65‐69. 16520512 10.1353/hpu.2006.0032

[58] Bradshaw M , Tomany‐Korman S , Flores G . Language barriers to prescriptions for patients with limited English proficiency: a survey of pharmacies. Pediatrics. 2007;120(2):e225‐e235. 17671036 10.1542/peds.2006-3151

[59] Hughes ML , John DN , Jones AT , Jones EH , Wilkins ML . Language issues in the community pharmacy: a perspective from Wales. Int J Pharm Pract. 2009;17(3):157‐163. 20218247

[60] Barnett CW , Muzyk AJ , Muzyk TL . Counseling Spanish‐speaking patients: Atlanta pharmacists' cultural sensitivity, use of language‐assistance services, and attitudes. J Am Pharm Assoc. 2004;44(3):366‐374. 10.1331/154434504323064002 15191247

[61] Mheidly N , Fares MY , Zalzale H , Fares J . Effect of face masks on interpersonal communication during the COVID‐19 pandemic. Front Public Health. 2020;8:898. 10.3389/fpubh.2020.582191 PMC7755855 33363081

[62] Valeriani G , Sarajlic Vukovic I , Lindegaard T , Felizia R , Mollica R , Andersson G . Addressing healthcare gaps in sweden during the COVID‐19 outbreak: on community outreach and empowering ethnic minority groups in a digitalized context. Paper presented at: Healthcare; 2020. 10.3390/healthcare8040445 PMC7712425 33139619

[63] Estacio EV , Oliver M , Downing B , Kurth J , Protheroe J . Effective partnership in community‐based health promotion: lessons from the health literacy partnership. Int J Environ Res Public Health. 2017;14(12):1550. 10.3390/ijerph14121550 PMC5750968 29232877

[64] Hajat C , Hasan A , Subel S , Noach A . The impact of short‐term incentives on physical activity in a UK behavioural incentives programme. NPJ Digit Med. 2019;2(1):91. 31531396 10.1038/s41746-019-0164-3 PMC6746750

[65] Lindegaard T , Brohede D , Koshnaw K , Osman SS , Johansson R , Andersson G . Internet‐based treatment of depressive symptoms in a Kurdish population: a randomized controlled trial. J Clin Psychol. 2019;75(6):985‐998. 30702758 10.1002/jclp.22753

[66] Noe TD , Kaufman CE , Kaufmann LJ , Brooks E , Shore JH . Providing culturally competent services for American Indian and Alaska Native veterans to reduce health care disparities. Am J Public Health. 2014;104(S4):S548‐S554. 25100420 10.2105/AJPH.2014.302140 PMC4151892

[67] Robinson A , Husband A , Slight R , Slight S . Digital technology to support lifestyle and health behaviour changes in surgical patients: systematic review. BJS Open. 2020;5(2)​. 10.1093/bjsopen/zraa009 PMC7944850 33688953

[68] Robinson A , Slight RD , Husband AK , Slight SP . Designing the optimal digital health intervention for patients' use before and after elective orthopedic surgery: qualitative study. J Med Internet Res. 2021;23(3):e25885. 33683208 10.2196/25885 PMC7985803

[69] Hanlon P , Daines L , Campbell C , McKinstry B , Weller D , Pinnock H . Telehealth interventions to support self‐management of long‐term conditions: a systematic metareview of diabetes, heart failure, asthma, chronic obstructive pulmonary disease, and cancer. J Med Internet Res. 2017;19(5):e172. 28526671 10.2196/jmir.6688 PMC5451641

[70] Bradford TW , Grier SA , Henderson GR . Weight loss through virtual support communities: a role for identity‐based motivation in public commitment. J Interact Mark. 2017;40:9‐23.

[71] van den Brand FA , Nagtzaam P , Nagelhout GE , Winkens B , van Schayck CP . The association of peer smoking behavior and social support with quit success in employees who participated in a smoking cessation intervention at the workplace. Int J Environ Res Public Health. 2019;16(16):2831. 10.3390/ijerph16162831 PMC6720923 31398854

[72] Bellamy C , Schmutte T , Davidson L . An update on the growing evidence base for peer support. Ment Health Soc Incl. 2017;25(5): 358‐364.

[73] Sahni H , Sharma H . Role of social media during the COVID‐19 pandemic: beneficial, destructive, or reconstructive? Int J Acad Med. 2020;6(2):70.

[74] Rossini P , Stromer‐Galley J , Baptista EA , Veiga de Oliveira V . Dysfunctional information sharing on WhatsApp and Facebook: the role of political talk, cross‐cutting exposure and social corrections. New Media Soc. 2021;23(8):2430‐2451.

[75] Armaou M , Araviaki E , Musikanski L . eHealth and mHealth interventions for ethnic minority and historically underserved populations in developed countries: an umbrella review. Int J Commun Well‐Being. 2020;3(2):193‐221.

[76] McCalman J , Jongen C , Bainbridge R . Organisational systems' approaches to improving cultural competence in healthcare: a systematic scoping review of the literature. Int J Equity Health. 2017;16(1):78. 28499378 10.1186/s12939-017-0571-5 PMC5429565

[77] Latif A , Waring J , Chen L‐c , et al. Supporting the provision of pharmacy medication reviews to marginalised (medically underserved) groups: a before/after questionnaire study investigating the impact of a patient–professional co‐produced digital educational intervention. BMJ Open. 2019;9(9):e031548. 10.1136/bmjopen-2019-031548 PMC6756439 31530620

[78] Latif A , Waring J , Pollock K , et al. Towards equity: a qualitative exploration of the implementation and impact of a digital educational intervention for pharmacy professionals in England. Int J Equity Health. 2019;18(1):1‐11. 31604434 10.1186/s12939-019-1069-0 PMC6790050

[79] Wenger LM , Rosenthal M , Sharpe JP , Waite N . Confronting inequities: a scoping review of the literature on pharmacist practice and health‐related disparities. Res Social Adm Pharm. 2016;12(2):175‐217. 26119111 10.1016/j.sapharm.2015.05.011

[80] White‐Means S , Dong Z , Hufstader M , Brown LT . Cultural competency, race, and skin tone bias among pharmacy, nursing, and medical students: implications for addressing health disparities. Med Care Res Rev. 2009;66(4):436‐455. 19369696 10.1177/1077558709333995

[81] Duckett K *. Pharmacy practice in hyperdiverse, urban communities: perspectives of independent community pharmacists in East and South‐East London*, London School of Hygiene & Tropical Medicine; 2011.

[82] Youmans SL , Schillinger D , Mamary E , Stewart A . Older African Americans' perceptions of pharmacists. Ethn Dis. 2007;17(2):284. 17682360

[83] Aspden T , Butler C , Moore B , Sheridan J . New Zealand health disparities–pharmacists' knowledge gaps and training needs. J Prim Health Care. 2011;3(3):192‐196. 21892420

[84] Tang W , Malone D . The relationship between medical insurance coverage and medication payments in the us from 1997 to 2015. Value Health. 2018;21:S90.

[85] National Institute for Health Research (NIHR) . *Toolkit for increasing participation of BAME groups in health and social care research*. 2018. Accessed February 1, 2022. https://arc-nenc.nihr.ac.uk/resources/toolkit-for-increasing-participation-of-bame-groups-in-health-and-social-care-research/

[86] National Institute for Health Research (NIHR) . *Improving inclusion of under‐served groups in clinical research: Guidance from INCLUDE project*. August 2020. Accessed February 1, 2022. https://www.nihr.ac.uk/documents/improving-inclusion-of-under-served-groups-in-clinical-research-guidance-from-include-project/25435

[87] Greenhalgh T , Wherton J , Shaw S , Morrison C . Video consultations for covid‐19. BMJ. 2020;368:m998. 32165352 10.1136/bmj.m998

[88] Archibald MM , Ambagtsheer RC , Casey MG , Lawless M . Using Zoom videoconferencing for qualitative data collection: perceptions and experiences of researchers and participants. Int J Qual Methods. 2019;18:1609406919874596.

[89] Lobe B , Morgan D , Hoffman KA . Qualitative data collection in an era of social distancing. Int J Qual Methods. 2020;19:1609406920937875.

[90] Joshi A , Bloom DA , Spencer A , Gaetke‐Udager K , Cohan RH . Video interviewing: a review and recommendations for implementation in the era of COVID‐19 and beyond. Academic Radiol. 2020;27(9):1316‐1322. 10.1016/j.acra.2020.05.020 PMC7833741 32563558

[91] Nielsen DS , Abdulkadir LS , Lynnerup C , Sodemann M . ‘I had to stifle my feelings’–Bilingual health professionals translating for family members in a healthcare setting. A qualitative study. Scand J Caring Sci. 2020;34(4):929‐937. 31830311 10.1111/scs.12800

[92] Seidelman RD , Bachner YG , eds. That I won't Translate! Experiences of a Family Medical Interpreter in a Multicultural Environment. Vol. 77. Wiley Online Library; 2010:389‐393. 10.1002/msj.20189 20687185

